# Exploration of the structure and inter­actions of 4-(di­methyl­amino)-3-methyl­phenyl *N*-methyl­car­bam­ate (Aminocarb)

**DOI:** 10.1107/S205322962500378X

**Published:** 2025-05-13

**Authors:** Oluwatoyin Akerele, Andreas Lemmerer

**Affiliations:** a Jan Boeyens Structural Chemistry Laboratory, Molecular Sciences Institute, School of Chemistry, University of the Witwatersrand, Private Bag 3, Johannesburg, 2050, South Africa; University of Oxford, United Kingdom

**Keywords:** crystal structure, noncovalent inter­actions, density functional theory, DFT, Amino­carb, synthetic pesticide

## Abstract

Amino­carb [4-(di­methyl­amino)-3-methyl­phenyl *N*-methyl­car­bam­ate, C_11_H_16_N_2_O_2_], a synthetic pesticide, was crystallized and characterized by single-crystal and powder X-ray diffraction. In the solid state, the mol­ecules have a strong chain N—H⋯O hy­dro­gen bond with a strength of −29.37 kJ mol^−1^ and a short C—H⋯π contact that build a wave-like three-dimensional structure. The structural stability and inter­molecular inter­action of Amino­carb were investigated using differential scanning calorimetry (DSC) and density functional theory (DFT). The results show that the com­pound is chemically stable, and the two dominating inter­actions are electrostatic and dispersion energies. An electrostatic potential map reveals the binding sites of the mol­ecules for reactivity. The understanding of the structural stability and interactions in Aminocarb provided in this study could be used to design new compounds with improved solubility and bioavailability. The dimethylamino group and the methyl group on the carbamate could be modified with other alkyl groups, which might reduce the Aminocarb toxicity, thereby leading to the development of safe, efficient and cost-effective compounds.

## Abstract 2

Aminocarb, a synthetic pesticide, was crystallized and characterized by single-crystal and powder X-ray diffraction. The structural stability and intermolecular interactions were investigated using differential scanning calorimetry (DSC) and density functional theory (DFT). The results show that the compound is chemically stable, and the two dominating interactions are electrostatic and dispersion energies.

## Introduction

Amino­carb is a synthetic pesticide in the class of car­bam­ates (Scheme 1). It is one of the most common classes of car­bam­ate that are commonly used for pest control of lepidopterous larvae, aphids, soil mollusks and other types of chewing insects. However, it is highly toxic to humans, animals and the environment (Moreira *et al.*, 2024 ▸; Dias *et al.*, 2014 ▸).
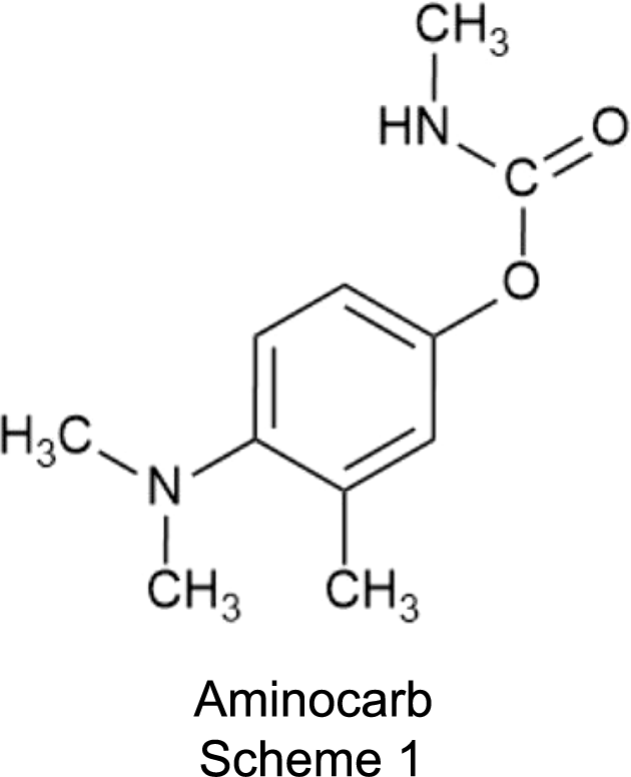


The structure of a mol­ecule and the inter­actions in the solid state are related to the properties of the com­pound. Therefore, insight into the structural characteristics of a com­pound could facilitate the modification of such a com­pound for optimal functionality and the elimination of toxicity. In fact, an understanding of the structural properties of a com­pound is important and has been used in different fields, including materials science, pharmaceuticals and agrochemicals (Choi, 2024 ▸; Biswas *et al.*, 2022 ▸), to improve the performance, solubility, bioavailability and stability properties of the material.

Studies have analysed the crystal structure of car­bam­ates to understand the chemical structures and properties (Xu *et al.*, 2005 ▸; Wu *et al.*, 2009 ▸; Xia, 2010 ▸), for instance, analysis of the ethyl car­bam­ate crystal structure provides insight into the geometry, noncovalent inter­actions and packing arrangements in the solid state (Bamigboye *et al.*, 2021 ▸). Similarly, the crystal structure of the inner salt of 2-[(amino­imino­meth­yl)amino]­ethyl­carbamic acid and its analysis highlighted the geometry and inter­actions in the com­pound (Matulková *et al.*, 2017 ▸). However, there is no study on the analysis of the crystal structure and properties of Amino­carb in the literature.

In this article, we will analyse the crystal structure of Amino­carb and its inter­actions to provide insight into the geometry, bonding, packing arrangement and stability of the structure in the solid state. Insecticides with better chemical stability last longer and are more effective in controlling pests and disease vectors. The insight from this study could facilitate the development of effective, safe and less toxic analogues of Amino­carb, thereby reducing the environmental pollution of the com­pound.

## Materials and methods

### Experimental

#### Crystal growth experiment

High-purity Amino­carb was purchased from Sigma–Aldrich and utilized as received. Amino­carb (10 mg) was dissolved in acetone (2 ml). The solution was heated and stirred gently on a hotplate at a low tem­per­a­ture of about 30 °C until the com­pound dissolved com­pletely. The heated solution was cooled to room tem­per­a­ture, before the vial was covered with a perforated Parafilm sheet to allow for the slow evaporation of the solvent. Prismatic crystals formed after 3 d and a suitably sized crystal was carefully selected for single-crystal X-ray diffraction (SCXRD) to ascertain the crystal structure.

#### Refinement

Crystal data, data collection and structure refinement details are summarized in Table 1 ▸. All atoms were refined anisotropically before the inclusion of H atoms. C-bound H atoms on aromatic rings were placed in calculated positions, and the *sp*^3^-hybridized C-bound H atoms were placed in calculated positions that match the electron-density map. The N-bound H atom was located in the difference Fourier map, and its fractional coordinates and isotropic displacement parameter refined freely.

#### Powder X-ray diffraction (PXRD)

A diffractogram of a powdered crystalline sample of Amino­carb was measured at 293 K using a Bruker D2 phaser powder X-ray diffractometer. The instrument was equipped with a sealed-tube Co *K*α_1_X-ray source (λ = 1.78896 Å) and a LynxEye PSD detector in Bragg–Brentano geometry, and operated at 30 kV and 10 mA. The data collection was carried out with a scanning inter­val ranging from 2θ = 5.0015 to 40° at a scan speed of 0.5 s per step (with an increment step size of 0.028445°). The *X’Pert High Score Plus* (Degen & van den Oever, 2009 ▸) software program was used to analyze the PXRD pattern, and the result was com­pared with the simulated pattern obtained from the SCXRD data to further establish the formation of the crystal structure.

#### Thermal analysis

Differential scanning calorimetry (DSC) detects the thermal changes in a material relative to a reference by measuring the change in the heat flow of a sample as tem­per­a­ture or time changes (Newman & Wenslow, 2018 ▸). Samples are heated and cooled to determine the melting points and enthalpies, and to detect any phase transitions. A Mettler Toledo DSC 3 was used to collect the DSC data, and aluminium pans were placed under nitro­gen gas at a flow rate of 10 ml min^−1^. The tem­per­a­ture and energy calibrations were performed using pure indium (purity 99.9%, m.p. 156.6 °C, heat of fusion 28.45 J g^−1^) and pure zinc (purity 99.9%, m.p. 419.5 °C, heat of fusion 112 J g^−1^). At a heating or cooling rate of 10 °C min^−1^, the samples were heated from 25 to 155 °C and then cooled to 25 °C.

#### Cambridge Structural Database analysis

The CSD (Version 5.44; Groom *et al.*, 2016 ▸) was used to analyse the structure of Amino­carb.

### Theoretical studies (using density functional theory, DFT)

#### Geometry optimization

The *GAUSSIAN16* suite of programs (Frisch *et al.*, 2016 ▸) was used for the minimization of Amino­carb with the default Berny algorithm (Li & Frisch, 2006 ▸). Geometry optimization of the Amino­carb structure obtained from the solved crystal structure was calculated in the gas phase at the M06l functional (Wang *et al.*, 2017 ▸; Zhao & Truhlar, 2008*a* ▸) with the def2-TZVP basis set (Zhao & Truhlar, 2008*b* ▸) and incorporate Grimme’s D3 dispersion correction (Grimme *et al.*, 2010 ▸) for a proper description of the dispersion inter­actions. The frequency com­putation was additionally com­puted at the same theoretical level (M06l-D3/def2-TZVP) to ensure no imaginary frequency and to ascertain that the structure that was optimized is a universal minimal structure with the lowest energy. *Chemcraft* (Zhurko & Zhurko, 2015 ▸) software was used in this study to analyze and visualize the output generated from Gaussian calculations. The program extracts the relevant information and presents it in a clear way for easy understanding of the output.

#### Inter­action energy of units in Amino­carb crystallized structures

The H-atom positions of two mol­ecular structures related by an inversion symmetry operation, obtained from the crystal structures, were optimized separately in the gas phase at the same theoretical (M06l-D3/def2-TZVP) method because the H-atom positions from crystal structures are not accurately determined by X-ray crystallography. This was done to obtain the inter­action energy between the units in the crystal structure of Amino­carb, and it was calculated with the same theoretical method, taking the basis set superposition error (BSSE) into account with the counterpoise correction (Simon *et al.*, 1996 ▸; Boys & Bernardi, 1970 ▸; Ransil, 1961 ▸). The expression for the inter­action energy is given by 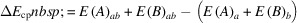
where Δ*E*_cp_ is the energy of com­plexation, *E*(*A*)_*ab*_ is the energy of *A* calculated in the presence of *B* ghost basis functions, similar to *E*(*B*)_*ab*_, *E*(*A*)_*a*_ is the energy of *A* and *E*(*A*)_*b*_ is the energy of *B*. The inter­action energy is 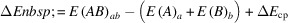


#### Hirshfeld surfaces and inter­molecular inter­action

*CrystalExplorer* (Spackman *et al.*, 2021 ▸) was used in this study to generate the Hirshfeld surfaces (HS) at high standard resolution using the CIF as the input file. *CrystalExplorer* creates colour-coded and HS surface maps that help to visualize the important regions of the inter­molecular inter­actions on the surface. The standard normalized contact (*d*_norm_) of Hirshfeld surface analysis is given as follows: 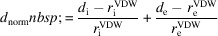
where *d*_i_ is the distance that represents nearest core inside the surface and *d*_e_ is the distance from the HS to the nearest core outside the surface (Dege *et al.*, 2022 ▸).

The *d*_norm_ is the distances range between the surface and the nearest atomic external surfaces (*d*_e_) and inter­nal surfaces (*d*_i_). Red contacts are those that are shorter than van der Waals radii (vdW), indicating that the atoms that form inter­molecular bonds are closer than the sum of their radii (Spackman *et al.*, 2008 ▸). Contacts with distances equal to the sum of the vdW radii are shown on the white surface. A blue colour indicates inter­actions that are more distinct – that is, contacts that are longer than the sum of the vdW radii (Dege *et al.*, 2022 ▸; Zeng *et al.*, 2023 ▸; Garg & Azim, 2022 ▸; Garg *et al.*, 2021 ▸, 2022 ▸).

*CrystalExplorer* was also used in this study to calculate the inter­molecular inter­action energies of the crystal structure. The wavefunctions of the mol­ecular system were calculated with the built-in *TONTO* program at the CE-B3LYP/6-31G(d,p) theoretical level (Jayatilaka & Grimwood, 2003 ▸; Mackenzie *et al.*, 2017 ▸; Turner *et al.*, 2015 ▸). All the energies of inter­action between the selected mol­ecule (at the centre of the cluster) and its neighbouring mol­ecules were com­puted; the model then separated the total energies into different com­ponents such as electrostatic, polarization, dispersion and repulsion energy com­ponents.

## Results and discussion

### Experimental results

#### Single-crystal X-ray diffraction (SCXRD) results

**Figure 1:**
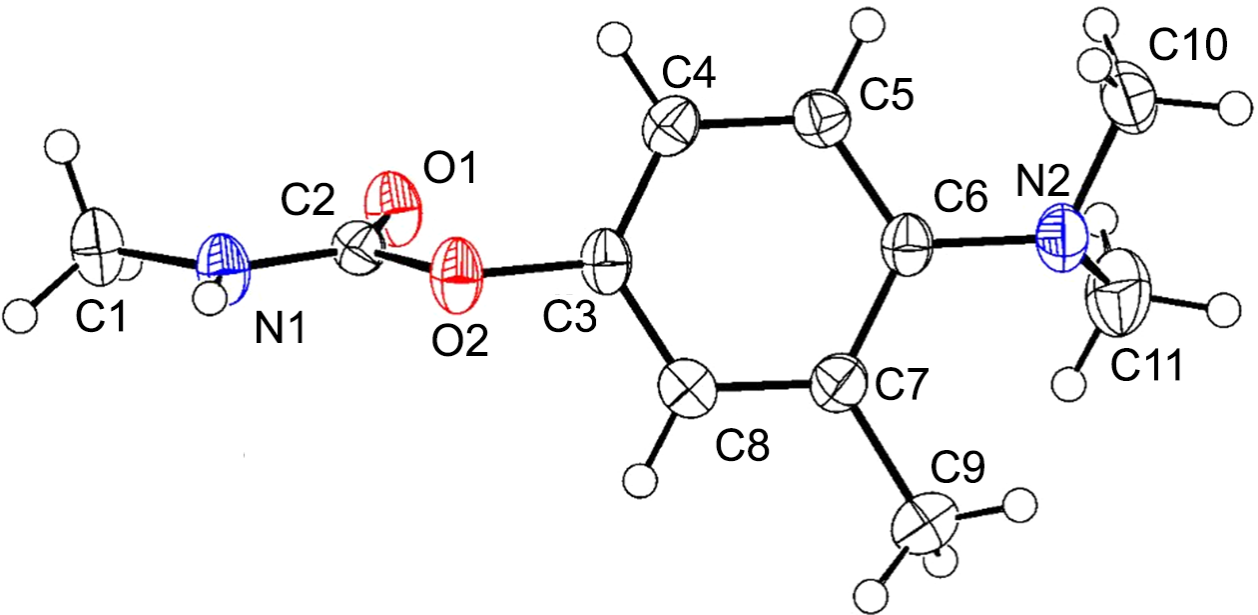
The mol­ecular structure and asymmetric unit of Amino­carb, with displacement ellipsoids drawn at the 50% probability level and H atoms shown as small spheres of arbitrary radii.

**Figure 2:**
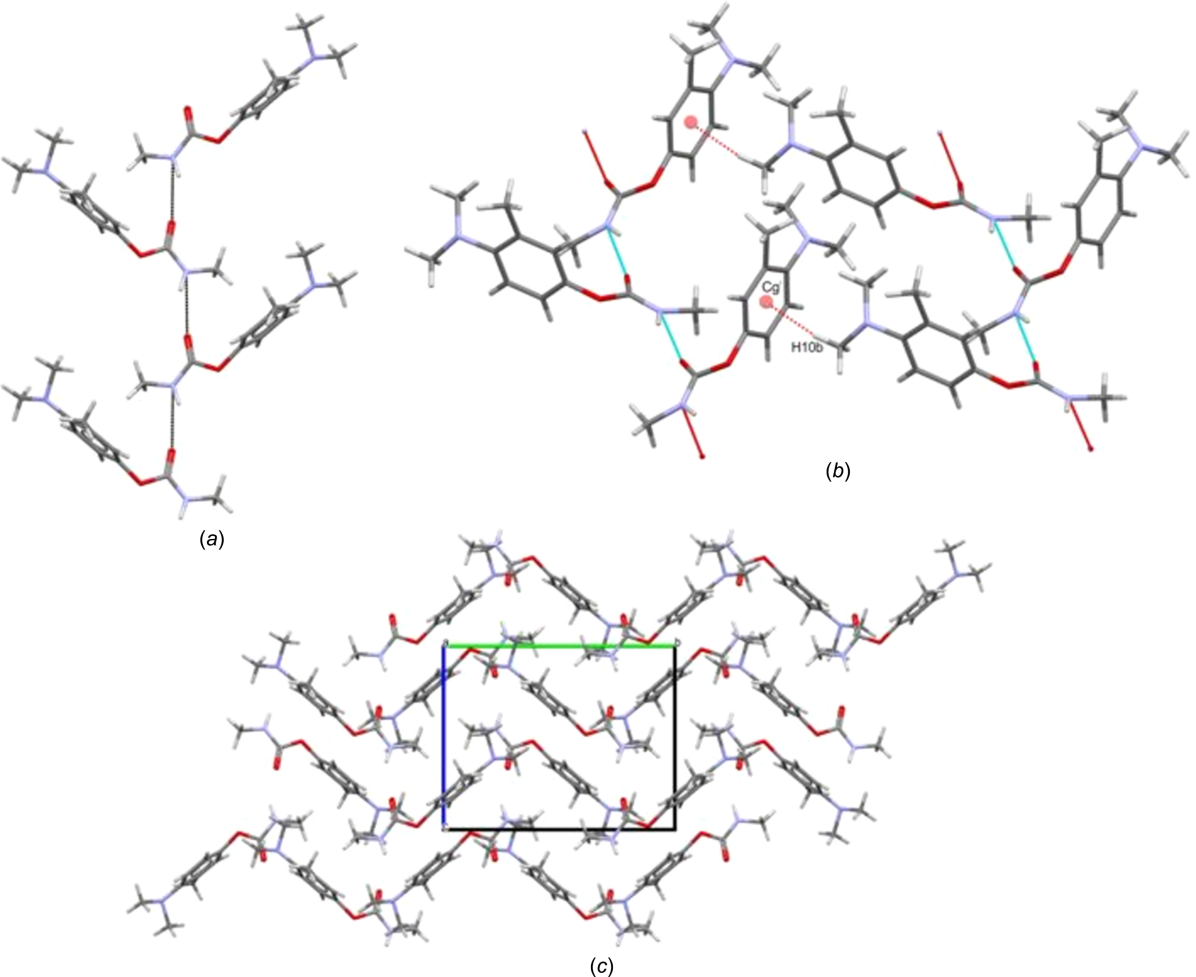
(*a*) The chain hy­dro­gen bond *C*(4); (*b*) the C—H⋯π contact joining adjacent chains [symmetry code: (i) *x*, −*y* + 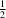, *z* − 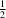]; (*c*) mol­ecules packed in opposite directions in a wave-like manner in the overall packing.

Amino­carb crystallized in the monoclinic space group *P*2_1_/*c* with *Z*′ = 1 (Fig. 1 ▸). Mol­ecules of Amino­carb related by the glide-plane operation form a hy­dro­gen-bonded chain with a *C*(4) motif (Bernstein *et al.*, 1995 ▸), using the N1—H1⋯O1^i^ hy­dro­gen bond, as shown in Fig. 2 ▸(*a*) and Table 2 ▸. Adjacent chains are connected *via* a short contact from H10*B* to the ring centroid of an adjacent mol­ecule [C10—H10*B*⋯π, with H10*B*⋯π = 2.90 Å; the centroid (*Cg*) is at (*x*, −*y* + 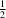, *z* − 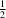)]. The Amino­carb mol­ecules form layers that stack on top of one another, giving a wave-like shape in the overall packing when viewed along the *a* axis, as shown Figs. 2 ▸(*b*) and 2(*c*).

#### PXRD

PXRD is an effective detection tool that can be used to ascertain phase purity in a bulk polycrystalline sample. The overlay of the powder pattern from the PXRD experiment and that simulated from the SCXRD experiment exhibit a shift in peak positions due to the different tem­per­a­tures of the experiments (293 and 173 K, respectively). The PXRD data are not of sufficient quality to conclusively rule out minor impurities, but the strong diffraction peaks are observed at related d-spacings in both patterns. The PXRD result is presented in the supporting information.

#### Differential scanning calorimetry (DSC)

**Figure 3:**
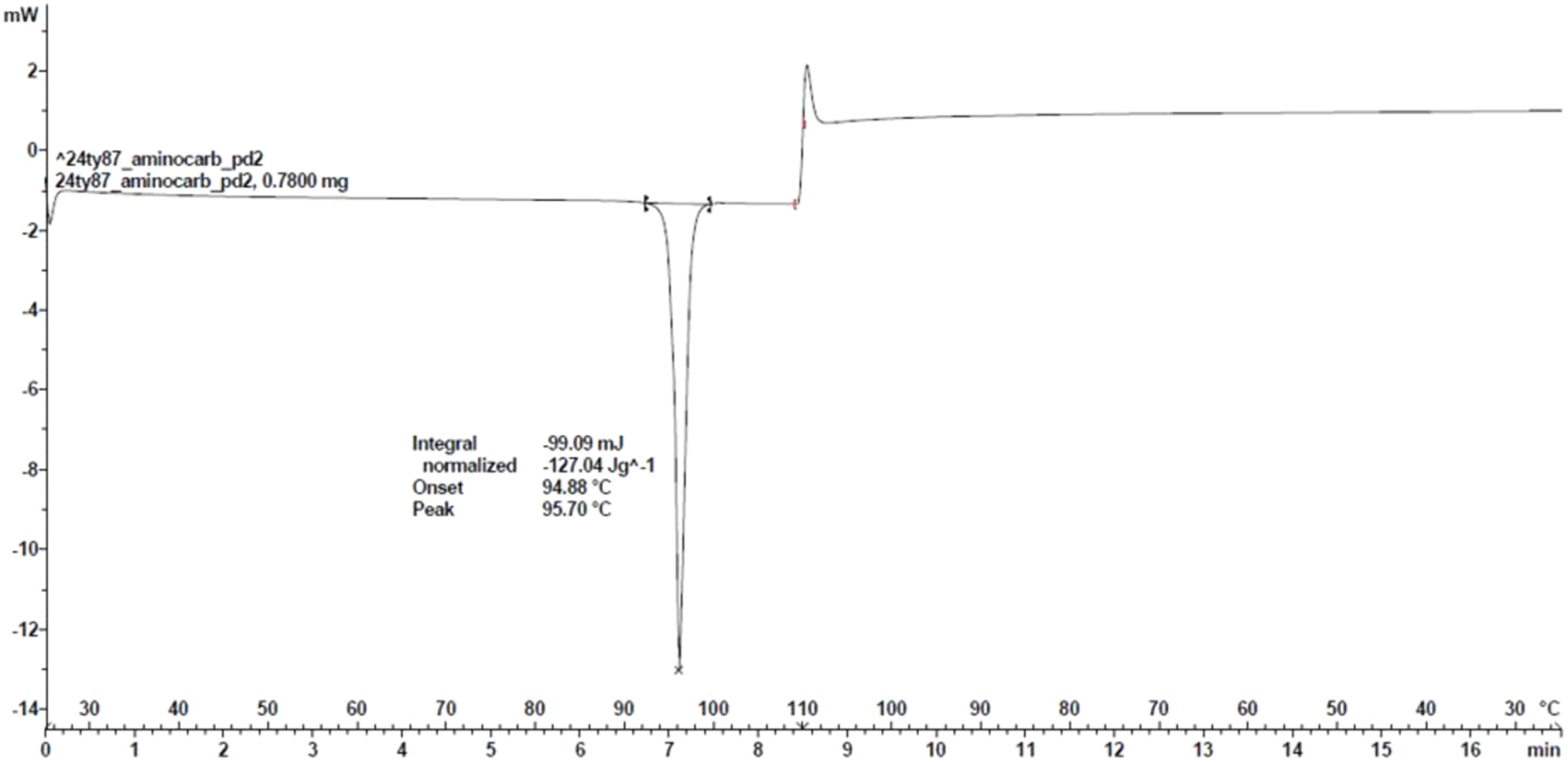
DSC scan of the Amino­carb structure.

The DSC thermograms of Amino­carb were obtained to determine the melting point and enthalpy of fusion, and to ascertain the presence of phase changes. The thermal data as given in Fig. 3 ▸ show an onset of melting point of 94.88 °C, and the enthalpy of melting is −26.46 kJ mol^−1^. The results indicate that Amino­carb is relatively thermally and chemically stable up to its melting point. However, the com­pound does not show any phase transition.

### Theoretical results

#### Full geometry optimization of the Amino­carb structure

**Figure 4:**
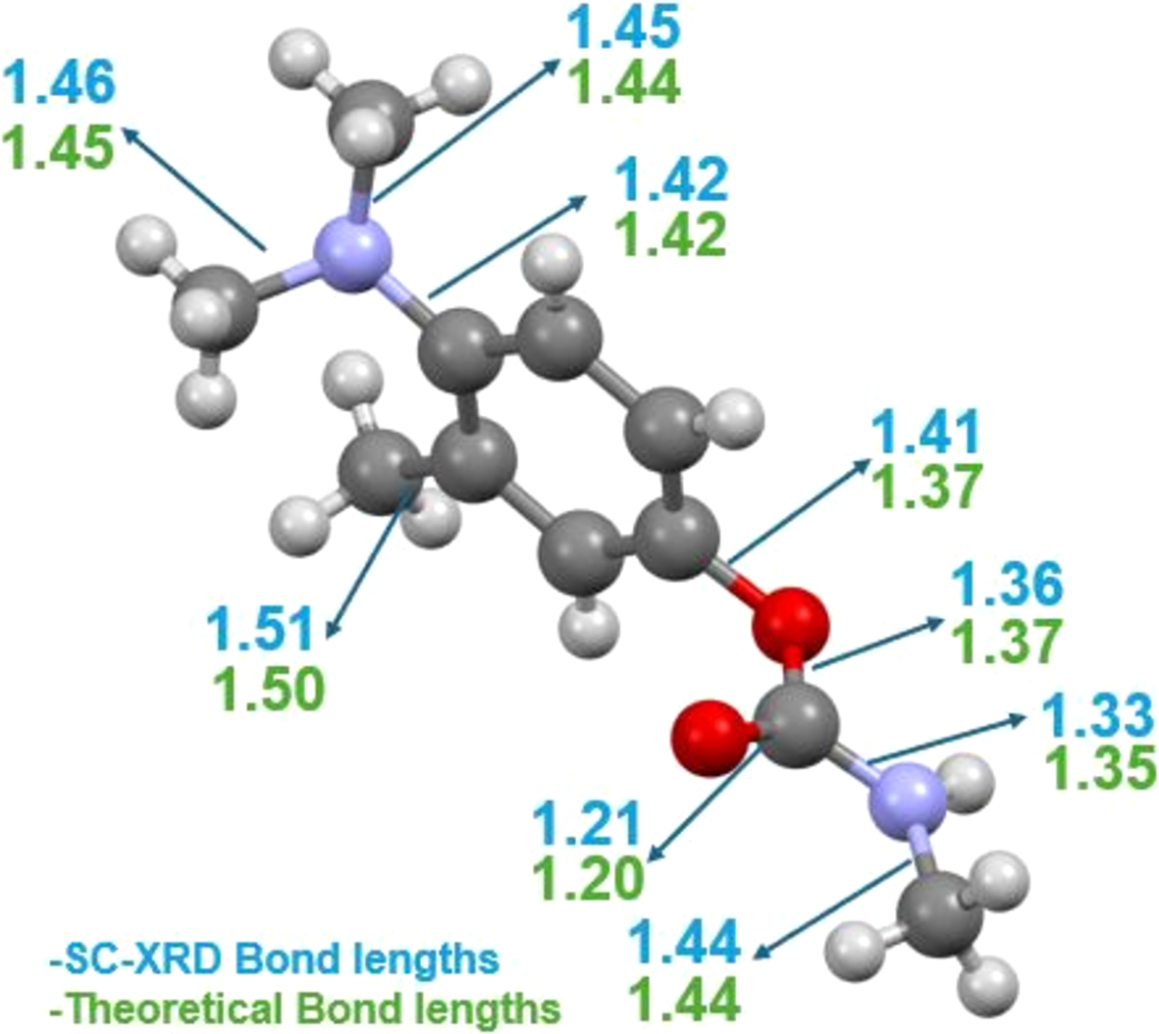
The geometry-optimized structure of Amino­carb. The experimental bond length is shown in sky blue, while the DFT bond length is in green (the stated values are in Ångstroms).

The optimized structure of Amino­carb (Fig. 4 ▸) calculated with density functional theory (DFT) has a minimum energy value of −1.81 × 10^6^ kJ mol^−1^. The geometry shows excellent agreement with the X-ray data of Amino­carb.

#### Energetic properties of the structure of Amino­carb

The inter­action energy between two Amino­carb structures related by a "glide plane operation is −7.02 kcal mol^−1^ (−29.37 kJ mol^−1^) in the gas phase. This suggests that the chain hy­dro­gen bond between two mol­ecules of Amino­carb, as shown in Fig. 2 ▸(*a*), is quite strong and is contributing to the stability of the crystal structure.

#### Hirshfeld surface (HS) analysis of the Amino­carb structure

To visualize the mol­ecular packing and inter­actions in the crystal structure, the structure HS analysis was generated using *CrystalExplorer* (Version 21) (Dege *et al.*, 2022 ▸; Spackman & Jayatilaka, 2009 ▸).

#### Fingerprint plots (FP)

**Figure 5:**
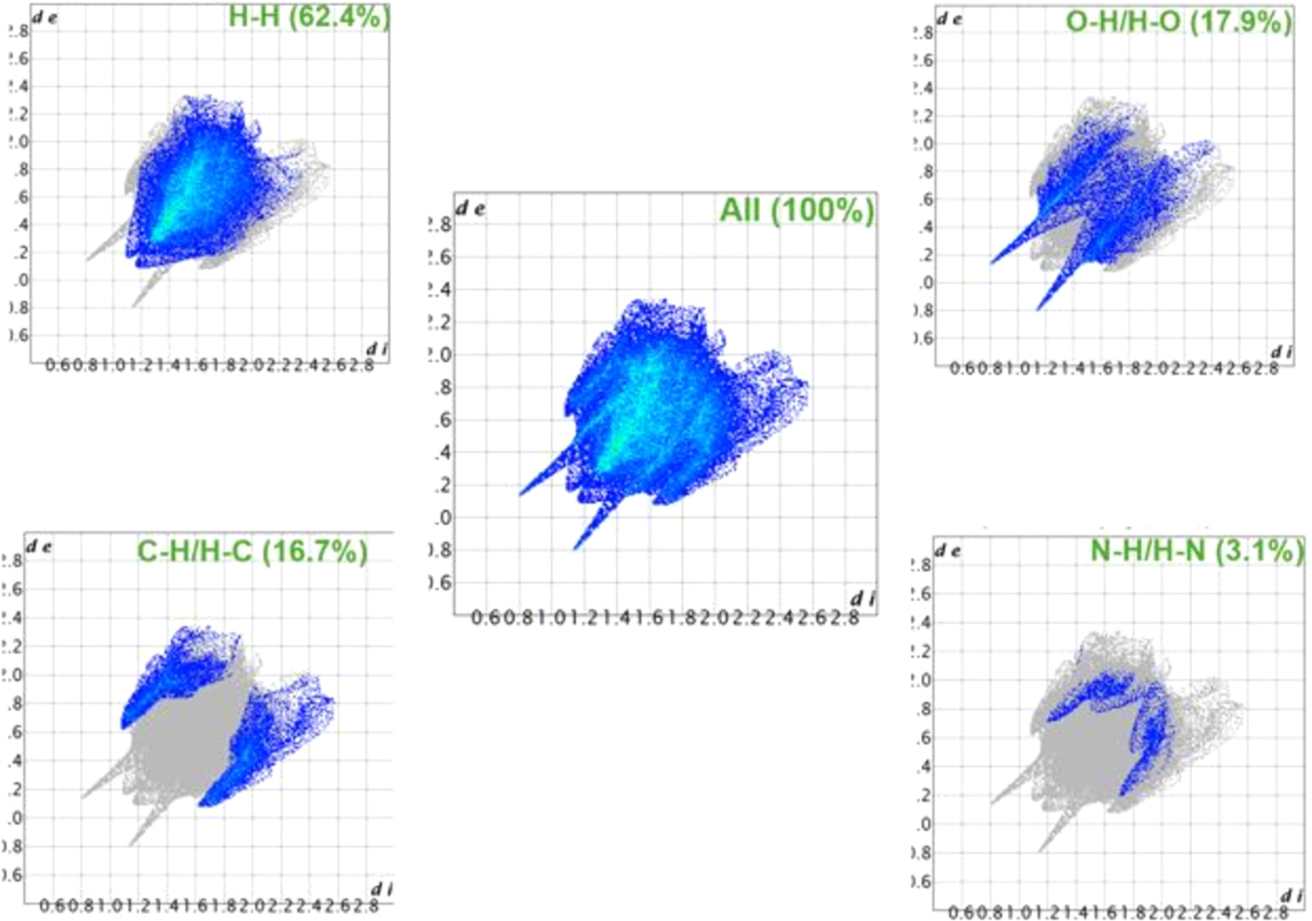
2D fingerprint plots of the Amino­carb structure.

The percentage contributions of the different atoms in close contact (inter­action) in the crystal structure packing of Amino­carb are given in Fig. 5 ▸. The *d*_i_ and *d*_e_ distances – the former representing the distance from the HS to the nearest atom outside and the latter representing the distance from the HS to the nearest atom in the inter­ior – are used to construct the FP (Dege *et al.*, 2022 ▸).

The 2D fingerprint plots of the structure, as well as split into individual elements, are displayed in Fig. 5 ▸. The H⋯H inter­actions account for the largest portion of the HS region in the 2D fingerprint maps, representing 62.4%. The O⋯H/H⋯O interactions account for 17.9% of the two sharp spikes. The other contacts are C⋯H/H⋯C (16.7%) and N⋯H/H⋯N (3.1%). These contacts suggest that electrostatic and dispersion inter­actions may be the dominant noncovalent inter­actions in the crystal structure.

#### Surfaces: *d*_norm_, curvedness, shape index and electrostatic potential

The colour-coded distances representing the different inter­molecular inter­actions of the structure were mapped onto the Hirshfeld surfaces.

**Figure 6:**
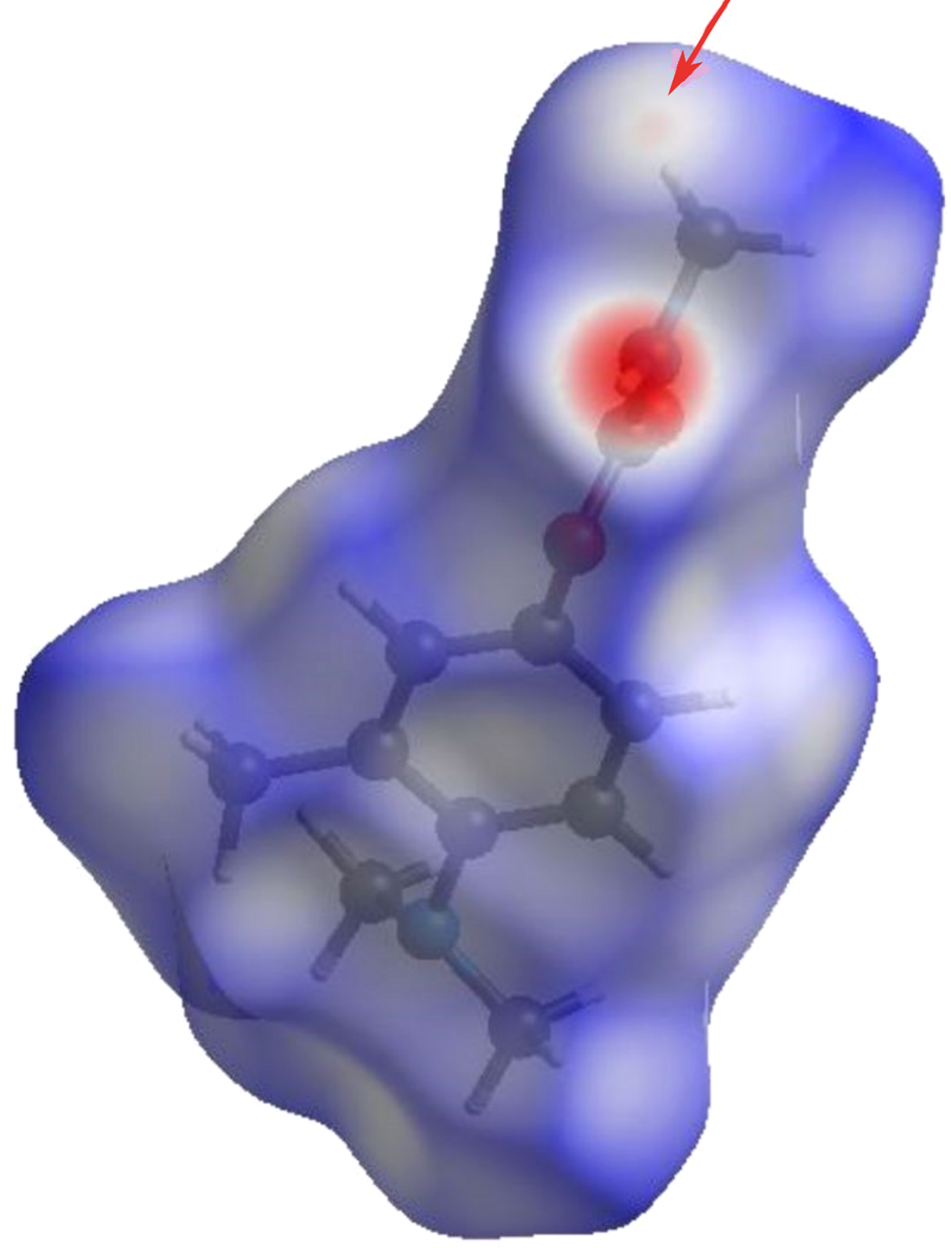
Three-dimensional Hirshfeld surface view along the *c* axis of Amino­carb mapped on *d*_norm_ between −0.5196 and 1.3010 a.u.

**Figure 7:**
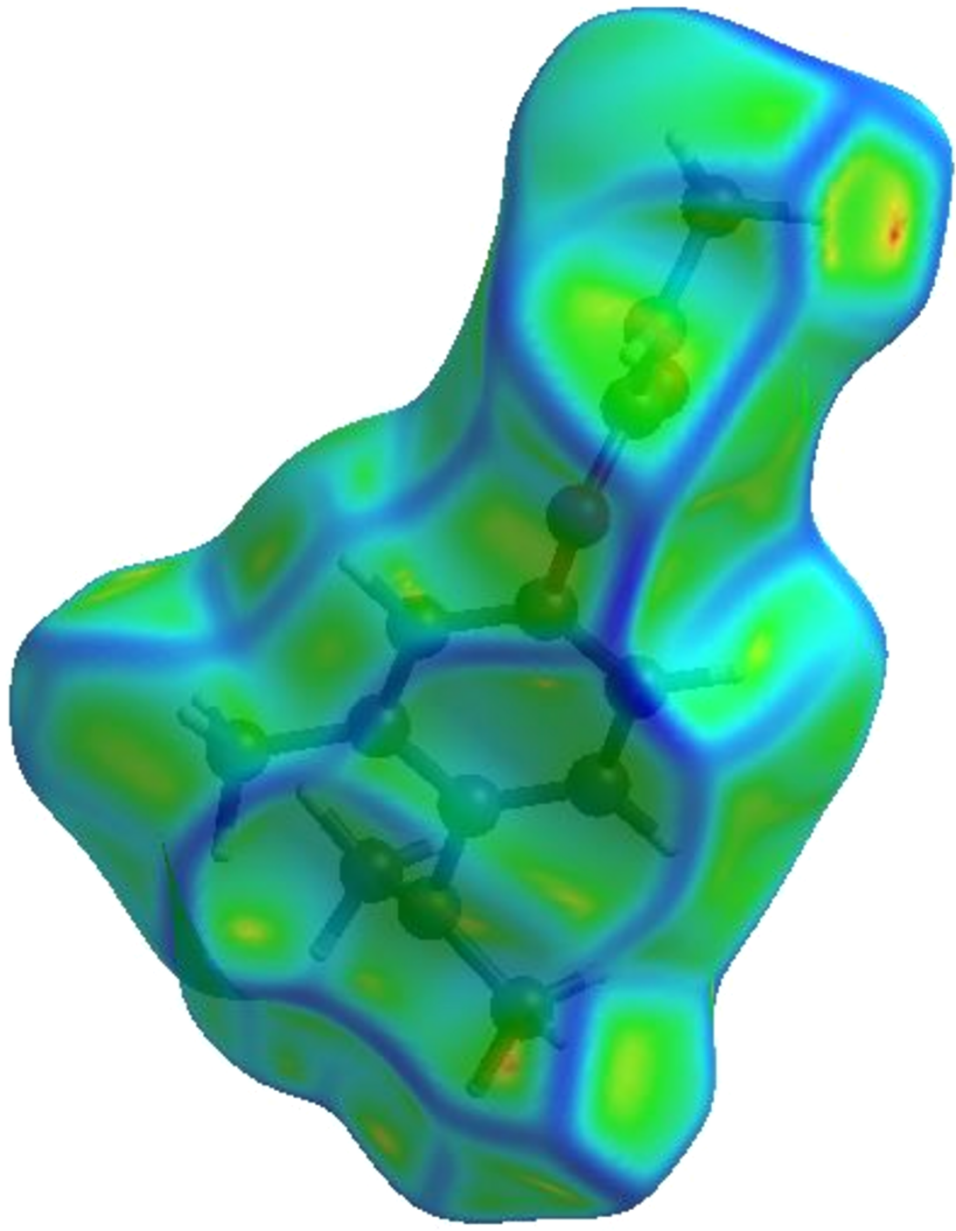
Three-dimensional Hirshfeld surface view along the *c* axis of Amino­carb mapped on curvedness between −4.0000 and 0.4000 a.u.

**Figure 8:**
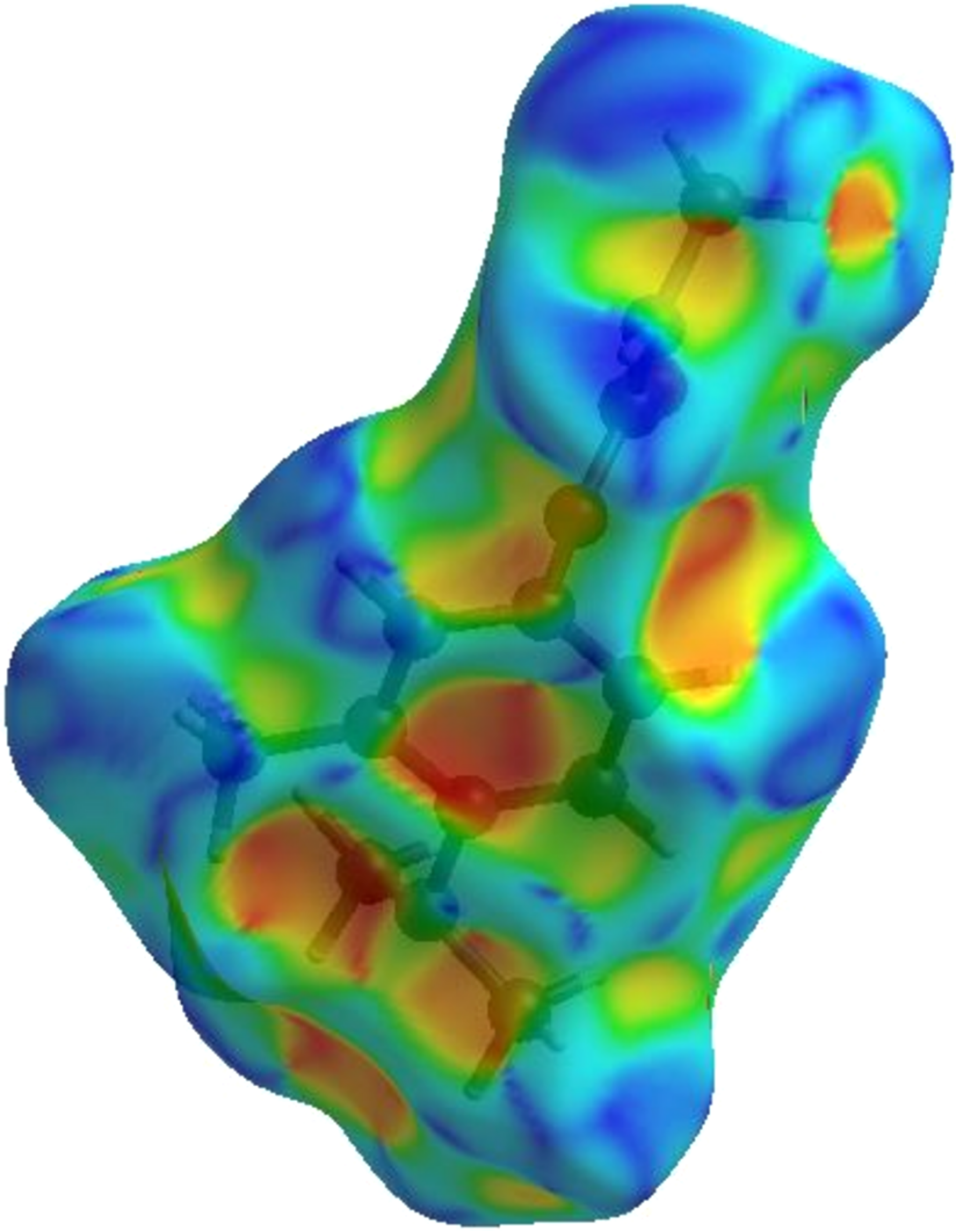
Three-dimensional Hirshfeld surface view along the *c* axis of Amino­carb mapped on shape index between −1.0000 and 1.0000 a.u.

**Figure 9:**
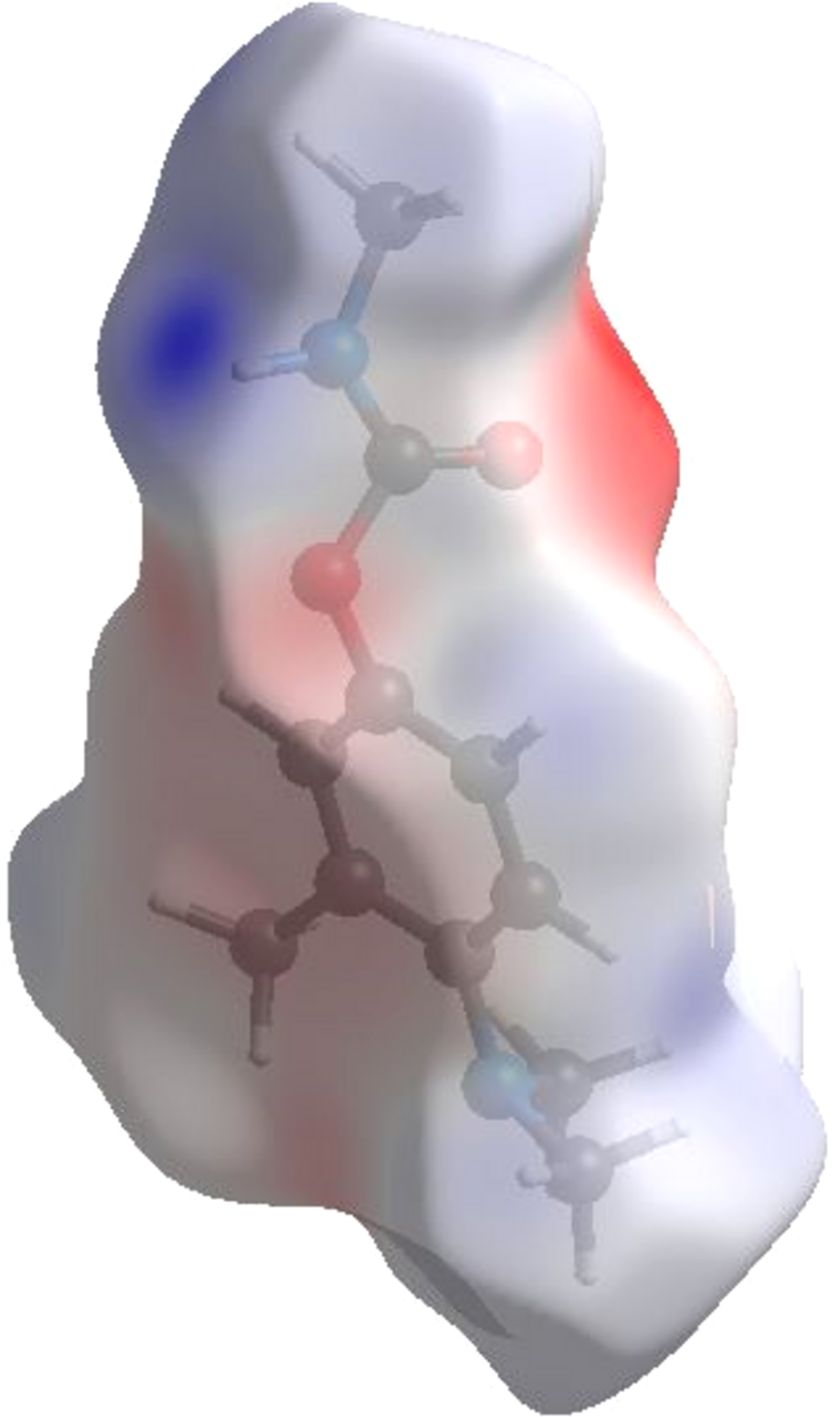
Three-dimensional Hirshfeld surface view of Amino­carb mapped on electrostatic potential between −0.0997 and 0.1443 a.u.

(1) The HS is plotted over *d*_norm_ in Fig. 6 ▸. The intensity of the red spots is a qualitative indicator of the strength of the contacts, as seen in the inter­molecular inter­actions presented. The N—H⋯O hy­dro­gen bonds produce two intense red patches, while the C—H contacts are indicated by a light-red region.

(2) A deeper understanding of mol­ecular packing was ob­tained by mapping the HS over shape index and curvedness. This was used to gain insight into the weak inter­actions in the crystal packing configuration. There is no indication of stacking inter­actions between mol­ecules of Amino­carb, as shown by the curvedness plot (Fig. 7 ▸), which does not display any flat surface area. This is in correlation with the fingerprint plot.

(3) The absence of red and blue triangles on the shape-index surface of Amino­carb shown in Fig. 8 ▸ demonstrated that the structure did not have a π–π stacking inter­action, which supported the absence of C⋯C contacts in the fingerprint plot (Spackman & Jayatilaka, 2009 ▸; Garg *et al.*, 2022 ▸; Akhileshwari *et al.*, 2022 ▸).

(4) The preferred binding sites and the electron donors and acceptors were identified and visualized with the use of an electrostatic potential (ESP) map. With the B3LYP/6-311G(d,p) theoretical method, the ESP property was com­puted on the HS surface at high standard resolution, and the results were mapped over the com­puted ESP (Fig. 9 ▸) (Mackenzie *et al.*, 2017 ▸; Turner *et al.*, 2017 ▸). The areas around the atoms that correspond to electropositive and electronegative potentials are shown by blue and red, respectively, as hy­dro­gen-bond donors and acceptors. On the ESP surface, the red region represents electrophilic sites and are electron deficient, while the blue region represents nucleophilic sites and are electron rich. In the Amino­carb structure, the negative potential is around C2=O1, O2 and the C atoms in the arene ring, while the positive potential is concentrated over N1—H1 and N2.

#### Inter­action energy of the crystal structure

The addition of inter­action energy calculations in *CrystalExplorer* allows for the precise calculation of the intensity of inter­actions, which may be directly com­pared to the outcomes obtained from HS analysis.

**Figure 10:**
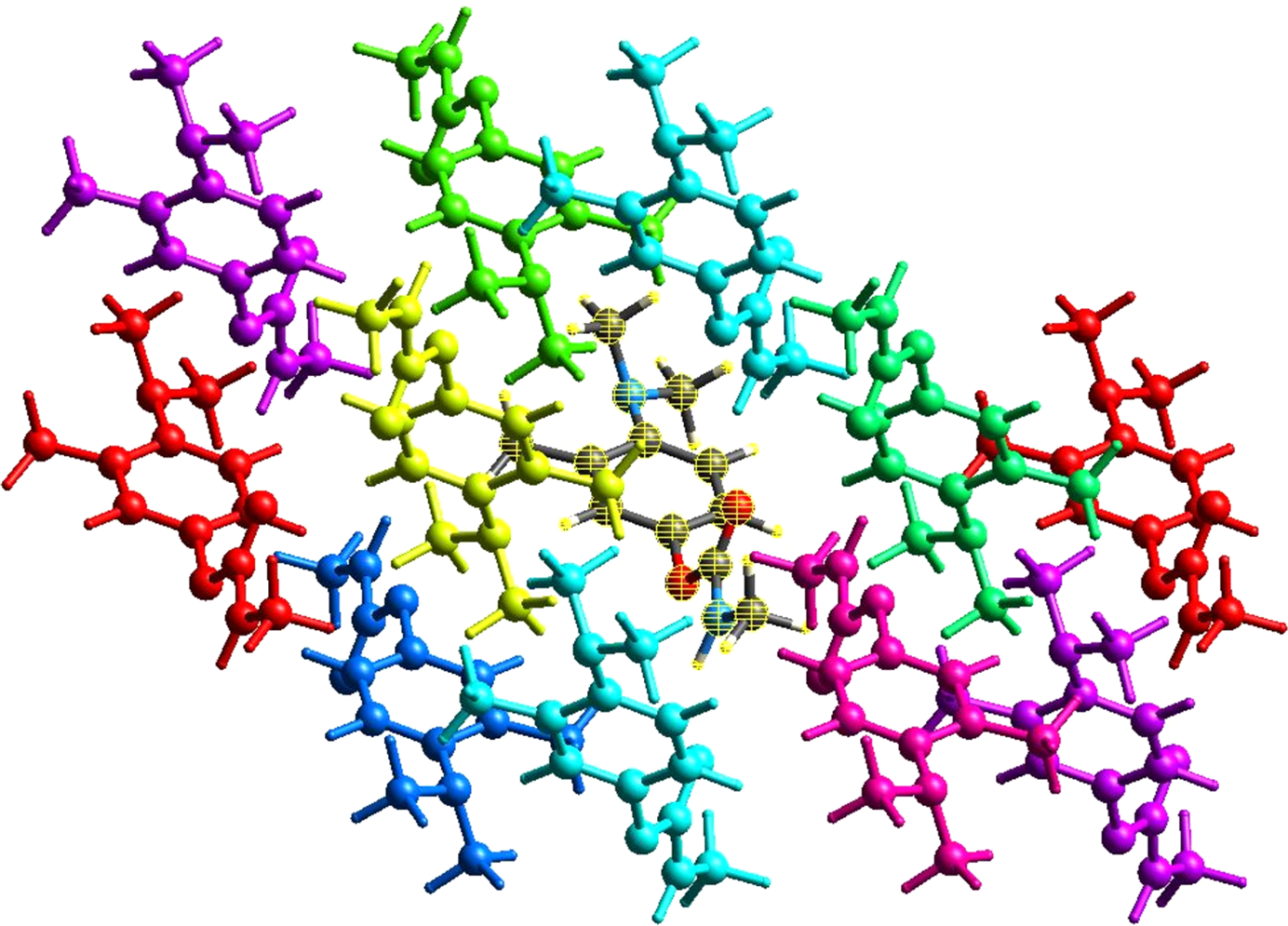
The Coulombic inter­action, dispersion and total inter­action energies of the Amino­carb mol­ecule are shown in red, blue, purple, green, yellow and pink along the *b* axis.

The CE-B3LYP/6-31G(d,p) energy model, which is accessible in *CrystalExplorer21* (Dege *et al.*, 2022 ▸), is used to com­pute the inter­molecular inter­action energies. By default, a cluster of mol­ecules is created by applying crystallographic symmetry operations to a chosen central mol­ecule within a radius of 3.8 Å (Frisch *et al.*, 1984 ▸). The total energies (*E*_tot_) are separated into different com­ponents, such as electrostatic (*E*_ele_), polarization (*E*_pol_), dispersion (*E*_dis_) and repulsion (*E*_rep_) energies (Hirshfeld, 1977 ▸), with their respective scale factors being 1.057, 0.740, 0.871 and 0.618 (Fig. 10 ▸).

The summation of energy of inter­actions (in kJ mol^−1^) for the Amino­carb structure (Table 3 ▸) are −54.75 (*E*_ele_), −11.91 (*E*_pol_), −108.53 (*E*_dis_), 57.17 (*E*_rep_) and −118.03 (*E*_tot_) for N—H⋯O. The evaluation of the energy com­ponents shows that the dispersion energy is the highest contributor to the stability of the structure. Electrostatic energy is also strong and contributes about half of the dispersion energy to the stability of the structure.

## Conclusions

Amino­carb crystallized in a monoclinic crystal system and the structure packed with a characteristic wave-like pattern. The geometry of the Amino­carb structure obtained experimentally is in excellent agreement with the theoretically optimized geometry. The chain hy­dro­gen bond in Amino­carb has an inter­action energy of −29.37 kJ mol^−1^, supporting the importance of this inter­action. DSC analysis shows that the structure is chemically stable, which is in correlation with the calculated inter­action energy. Reactive sites in Amino­carb are identified using a mol­ecular electrostatic potential map. The dominant contacts in the fingerprint plot are O⋯H, C⋯H and H⋯H, confirming the presence of electrostatic and dispersion energy as the dominant inter­action energies. The detailed information of the structural analysis, bonding, stability property and inter­actions energy in Amino­carb provided in this study could be used to modify the structure through functional-group manipulation to improve the properties and perhaps aid the development of safe and effective Amino­carb.

## Supplementary Material

Crystal structure: contains datablock(s) I, global. DOI: 10.1107/S205322962500378X/op3034sup1.cif

Structure factors: contains datablock(s) I. DOI: 10.1107/S205322962500378X/op3034Isup2.hkl

Overlay of simulated and experimental powder patterns. DOI: 10.1107/S205322962500378X/op3034sup3.pdf

PLATON output. DOI: 10.1107/S205322962500378X/op3034sup4.txt

Supporting information file. DOI: 10.1107/S205322962500378X/op3034Isup5.mol

Supporting information file. DOI: 10.1107/S205322962500378X/op3034Isup6.cml

CCDC reference: 2416073

Additional supporting information:  crystallographic information; 3D view; checkCIF report

## Figures and Tables

**Table 1:** Experimental details

Crystal data	
Chemical formula	C_11_H_16_N_2_O_2_
*M*_r_	208.26
Crystal system, space group	Monoclinic, *P*2_1_/*c*
Temperature (K)	173
*a*, *b*, *c* (Å)	9.2445 (3), 12.4193 (4), 9.9910 (4)
β (°)	98.929 (1)
*V* (Å^3^)	1133.17 (7)
*Z*	4
Radiation type	Mo *K*α
μ (mm^−1^)	0.09
Crystal size (mm)	0.29 × 0.28 × 0.15

Data collection	
Diffractometer	Bruker APEXII CCD
Absorption correction	Multi-scan (*SADABS*; Bruker, 2016 ▸; Krause *et al.*, 2015 ▸)
*T*_min_, *T*_max_	0.595, 0.746
No. of measured, independent and observed [*I* > 2σ(*I*)] reflections	20029, 2719, 2468
*R*_int_	0.056
(sin θ/λ)_max_ (Å^−1^)	0.660

Refinement	
*R*[*F*^2^ > 2σ(*F*^2^)], *wR*(*F*^2^), *S*	0.041, 0.118, 1.06
No. of reflections	2719
No. of parameters	145
H-atom treatment	H atoms treated by a mixture of independent and constrained refinement
Δρ_max_, Δρ_min_ (e Å^−3^)	0.34, −0.27

Computer programs: *APEX4* (Bruker, 2016 ▸), *SAINT* (Bruker, 2016 ▸), *SHELXT2018* (Sheldrick, 2015*a* ▸), *SHELXL2018* (Sheldrick, 2015*b* ▸), *OLEX2* (Dolomanov *et al.*, 2009 ▸) and *Mercury* (Macrae *et al.*, 2020 ▸).

**Table 2:** Hydrogen-bond geometry (Å, °)

*D*—H⋯*A*	*D*—H	H⋯*A*	*D*⋯*A*	*D*—H⋯*A*
N1—H1⋯O1^i^	0.825 (17)	2.083 (17)	2.8174 (12)	148.1 (15)

Symmetry code: (i) 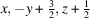.

**Table 3:** The theoretical inter­action energy of the Amino­carb mol­ecule (in kJ mol^−1^) The total of the four energy com­ponents – polarization (*E*_pol_), dispersion (*E*_dis_), repulsion (*E*_rep_) and electrostatic (*E*_ele_) energies – is the inter­action energy (*E*_tot_). *R* is the atomic position (distance in Å) between mol­ecular centres.
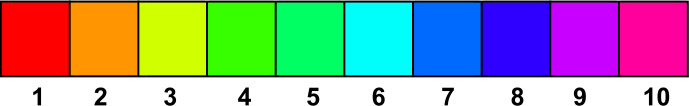

No.	*N*	Symop	*R*	Electron density	*E*_ele_	*E*_pol_	*E*_dis_	*E*_rep_	*E*_tot_
**1**	2	*x*, *y*, *z*	9.24	B3LYP/6-31G(d,p)	0.2	−0.1	−1.7	0.0	−1.3
**2**	2	*x*, −*y* + 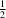 , *z* + 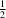	8.20	B3LYP/6-31G(d,p)	−5.3	−0.9	−24.2	15.0	−18.1
**3**	2	−*x*, *y* + 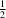 , −*z* + 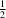	7.50	B3LYP/6-31G(d,p)	−3.0	−1.3	−26.2	14.6	−17.9
**4**	1	−*x*, −*y*, −*z*	6.81	B3LYP/6-31G(d,p)	−4.5	−1.8	−23.7	8.6	−21.4
**5**	2	−*x*, *y* + 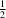 , −*z* + 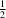	8.14	B3LYP/6-31G(d,p)	−6.7	−2.9	−16.5	10.9	−16.9
**6**	2	*x*, −*y* + 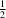 , *z* + 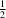	7.74	B3LYP/6-31G(d,p)	−34.2	−8.9	−16.5	37.6	−33.8
**7**	1	−*x*, −*y*, −*z*	6.25	B3LYP/6-31G(d,p)	−2.8	−1.0	−14.5	3.9	−13.9
**8**	1	−*x*, −*y*, −*z*	5.90	B3LYP/6-31G(d,p)	−1.2	−0.9	−29.9	16.1	−18.1
**9**	2	*x*, −*y* + 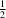 , *z* + 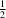	11.45	B3LYP/6-31G(d,p)	0.3	−0.0	−1.4	0.1	−0.9
**10**	1	−*x*, −*y*, −*z*	13.48	B3LYP/6-31G(d,p)	2.3	−0.3	−8.1	0.0	−4.9
