# Supplementary material for: Exploration of the structure and inter­actions of 4-(di­methyl­amino)-3-methyl­phenyl N-methyl­car­bam­ate (Aminocarb)
Source: Acta Crystallogr C Struct Chem. 2025 May 13;81(Pt 6):310–8. doi: 10.1107/S205322962500378X (PMC12138253; doi:10.1107/S205322962500378X)
Supplement: Supplementary file 3 [file c-81-00310-sup3.pdf]

STRUCTURAL CHEMISTRY

Volume 81 (2025)

Supporting information for article:

Exploration of the structure and interactions of 4-(dimethylamino)-3-methylphenyl *N*-methylcarbamate (Aminocarb)

Oluwatoyin Akerele and Andreas Lemmerer

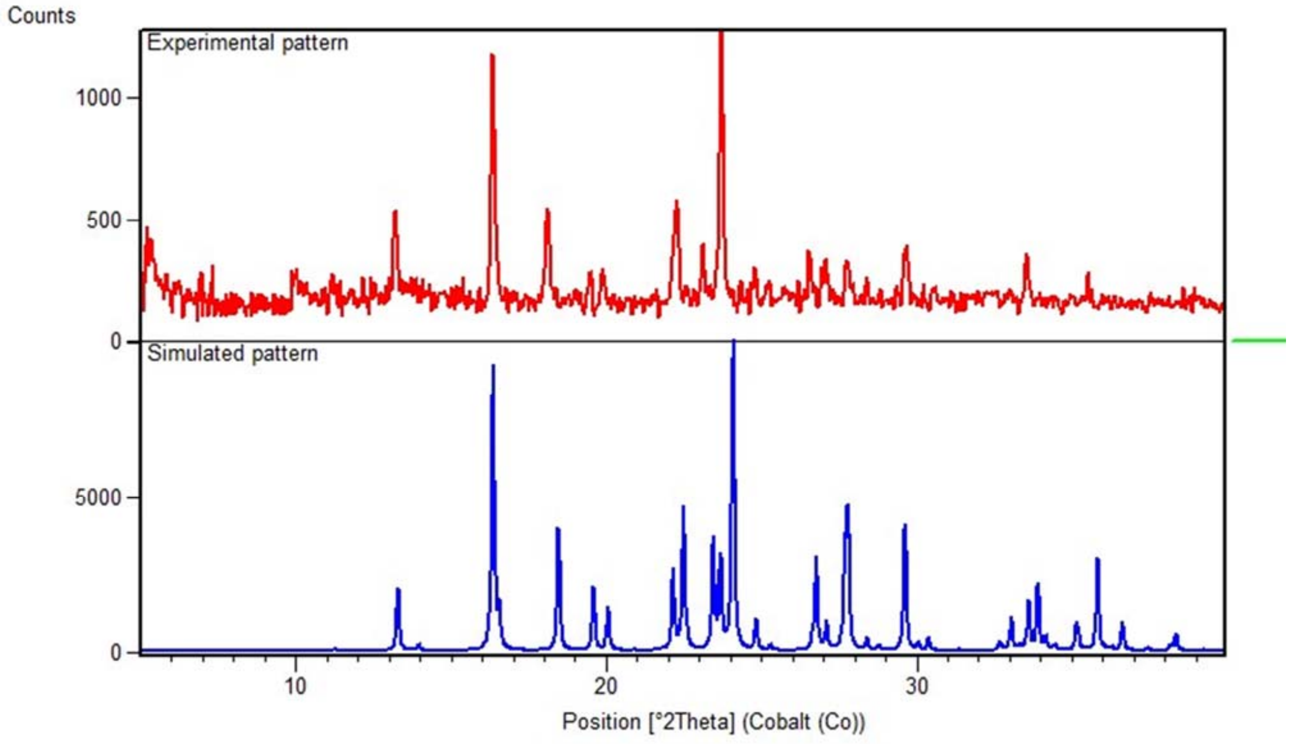

Fig. S1. The PXRD patterns for Aminocarb.
